# Knowledge about HIV in a Community Sample of Urban African Americans in the South

**DOI:** 10.4172/2155-6113.1000622

**Published:** 2016-10-08

**Authors:** H Klein, CE Sterk, KW Elifson

**Affiliations:** Department of Behavioral Sciences and Health Education, Rollins School of Public Health, Emory University Atlanta, Georgia

**Keywords:** HIV knowledge, African Americans, Urban, South

## Abstract

**Purpose:**

Race and HIV are intertwined in complex ways. African Americans, particularly those residing in the southern United States, are at great risk for contracting and subsequently transmitting HIV. Research on the extent to which members of this population understand the risks associated with engaging in specific behaviors is limited. This paper examines HIV knowledge among at-risk adult African American men and women and the factors associated with levels of HIV knowledge.

**Methods:**

Based on a conceptual model derived from Social Disorganization Theory and Syndemics Theory, interviews were conducted between 2009 and 2011. Questionnaire-based interviews were conducted with 1,864 respondents from 80 strategically-chosen census block groups in Atlanta, Georgia. An innovative approach to assessing amount of HIV knowledge was implemented, to derive better estimates of the extent of knowledge.

**Results:**

Overall, HIV knowledge was low (average=43.5% correct answers). Seven factors were identified as contributing uniquely to having higher levels of knowledge about HIV transmission: (1) younger age, (2) being educated beyond the high school level, (3) being gay, lesbian or bisexual, (4) experiencing sexual abuse during childhood and/or adolescence, (5) drinking alcohol less frequently, (6) knowing a larger number of HIV-infected persons and (7) knowing anyone currently living with “full blown” AIDS.

**Conclusion:**

HIV educational and intervention programs targeting at-risk African American adults need to develop effective ways of bolstering a solid understanding of how HIV is/not transmitted. In particular, efforts need to be targeted toward older adults, those with lower levels of educational attainment and persons who are not acquainted with anyone who is HIV-infected.

## Introduction

According to recent data reported by the Centers for Disease Control and Prevention, of the four geographic regions used to group states forming the United States for health monitoring purposes, the South has the highest rates of new infections of HIV and other sexually-transmitted infections (STIs) such as chlamydia, gonorrhea, and syphilis. For chlamydia, people residing in the South have infection rates that are 8.8% above the national average. For gonorrhea, the disparity is even greater, with an incidence rate that is 22.7% higher than the national average among Southerners. It is greater still for primary and secondary syphilis, with a 27.5% higher incidence rate, and even more disparate for HIV, for which Southerners have new infection rates that are 32.3% above the national average. Even within the states comprising the South, HIV and STI infection rates are not uniform. The state of Georgia (where the present research was conducted) reported the 5th highest rates of gonorrhea (18.3% higher than the South as a whole) and the 5^th^ highest rates of chlamydia (7.5% higher than the South as a whole) out of the 16 states classified as comprising the South. Georgia has the fourth highest rate of new HIV infections amongst the Southern states, with an incidence rate that is 50.2% higher than the South as a whole. Moreover, Georgia has the highest rates of primary and secondary syphilis in the nation–61.0% higher than the South as a whole and 90.0% higher than the nation as a whole. Even within the state of Georgia, HIV and STI infection rates are not uniform. For example, the Atlanta metropolitan area (where the present research was conducted) has rates of primary and secondary syphilis that are 46.3% higher than those for the state of Georgia as a whole, and rates of gonorrhea and chlamydia that are close to the state-wide average. Incidence rates for HIV within Atlanta mirror those for the state as a whole and are 45.0% higher than for the South overall [1]. Unmistakably, Atlanta is a geographic location that is experiencing high rates of sexual risk taking and, thus, is in need of effective education, prevention and intervention services to help combat the HIV and STI epidemics.

Complicating matters, within a particular region, HIV and STI infection rates also differ based on selected demographic characteristics. One of the most important differentiating factors is race/ethnicity, with people in communities of color being particularly hard hit by the HIV and STI epidemics. Among African Americans (who comprise the sample from which the present research was derived), primary or secondary syphilis rates are six times greater than they are among Caucasians. For chlamydia, the African American versus Caucasian racial disparity increases to seven times; and for gonorrhea, it increases to fifteen times. During the most recent reporting year, among people newly infected with HIV, African Americans had an incidence rate that was 8.6 times greater than Caucasians and 3.8 times greater than amongst people of all racial/ethnic backgrounds. African Americans accounted for 46.2% of all people newly diagnosed with HIV during the most recent reporting year, while comprising 13.1% of the adult U.S. population-at-large [2] (U.S. Census Bureau, 2014). Within the state of Georgia–earlier shown to be well above the national average for new HIV infections–African Americans comprised 69.6% of all new HIV infections during the most recent reporting year [1], even though they accounted for only 30.5% of the state’s adult population [3]. At-risk African Americans, particularly those living in the South, and especially those residing in Georgia, are clearly in need of effective education, prevention, and intervention services to help combat the HIV and STI epidemics.

The very high rates of HIV and STI infections among these individuals beg an important question: What factors account for the high rates of infections among members of this population? Over the years, a number of factors have been identified as contributing to these rates. For example, the influence of religiosity and in particular the influence of the Black Church on matters pertaining to sexual safety has been cited by some researchers as a key factor underlying HIV risk taking in the African American community. On this point, some researchers have demonstrated that, among African Americans, greater religiosity was associated with higher levels of stigma toward HIV/AIDS and with more prejudice against persons with HIV/AIDS [4]. Others have shown that greater religiosity was linked with lower knowledge about HIV and its transmission [5], with the authors attributing this, in part, to religiosity-induced HIV/AIDS stigma. The Black Church has also been accused of being too silent on matters pertaining to HIV/AIDS and HIV risk taking, and failing to provide essential leadership to its members, who often look to their faith leaders for guidance and helpful information about how to lead their lives [6,7].

Another explanation that has been offered to help explain the racial health disparities is sociocultural in nature and pertains to differences in gender roles, specifically, masculinity norms. Research findings have shown that African American men score higher than members of other racial/ethnic groups, especially Caucasians, on measures of masculinity [8,9] and that Caucasian and African American men define masculinity differently [10]. When applied to situations involving sexual behavior, men who score higher on masculinity have been shown to be more likely to engage in HIV sexual risk behaviors compared to their less-masculine counterparts [11–13]. Coinciding with this, norms supporting higher levels of masculinity make it more difficult for women to broach the subject of sexual safety with their male sex partners, either due to the perceived normative “inappropriateness,” the perceived unacceptability of raising this subject with their male partners and/or due to fear of reprisal, violence, and/or abandonment by those partners [14–16].

A third factor linking greater involvement in HIV sexual risk practices among African Americans is lower condom use self-efficacy. As the present authors have discussed in previous papers [17–19], condom use self-efficacy has three major components to it. The first of these is the ability to use condoms correctly and to have the skills necessary for their proper use, such as checking their packaging for the expiration date, safe removal of the device from its wrapper, proper lubrication and placement on the penis, and proper and safe removal of the device after use. The second component to condom use self-efficacy is making sure that users have condoms at their disposal whenever they want and need to use them, which includes elements such as knowing which type of condom(s) to use, where to purchase or obtain the condom(s) in question, and where to store them for ease of use. The third element of condom use self-efficacy is having the ability to discuss using condoms with one’s sex partner(s), feeling comfortable (or at the very least, not feeling too ill-at-ease) and confident in broaching the subject of using condoms with one’s sex partner(s), and to negotiate successfully with one’s sex partners(s) for their use. In the scientific literature, evidence has been accumulating to suggest that various subgroups of African American adults may have lower overall levels of condom use self-efficacy, thereby elevating their risk for contracting HIV and other STIs. This has been found to be true for African Americans who are crack users [20], homeless [21], adolescent females [22], juvenile offenders [23], rural-dwelling women aged 50 or older [24] and HIV-positive men who have sex with men [25].

A fourth factor that has been cited as accounting for racial health disparities among African Americans is HIV/AIDS knowledge–specifically, that many members of this population are not well-informed about HIV and how it is (and how it is not) transmitted [26–30]. Studies examining the nexus of HIV knowledge and involvement in HIV risk behaviors have yielded inconsistent findings [30–34], which generally has led researchers to conclude that increasing HIV knowledge is insufficient as an intervention for reducing HIV risk behaviors. Nevertheless, we cannot reasonably expect people to change behaviors that they do not know may cause them harm. Therefore, even if it bolstering HIV knowledge is not sufficient to reduce HIV risk involvement, it nevertheless remains a necessary step in the risk-reduction process.

In the present paper, we focus our attention on the latter subject–namely, HIV knowledge–in a community sample of urban, Southern-dwelling African American adults. Using an innovative method of assessing HIV knowledge, we examine the following research questions: (1) What level of knowledge do members of the target population have with regard to HIV and HIV transmission? (2) What specific gaps in HIV knowledge exist in this target population? (3) What factors are associated with greater knowledge about HIV? We conclude the paper by discussing the education, prevention, and intervention implications of these findings.

## Methods

### Procedures

Data for this study were collected as part of People and Places, a cross-sectional study of people’s perceptions of how their neighborhood impacted their daily lives and actions. Data were collected between May 2009 and August 2011 in Atlanta, Georgia. Participants (n=1,864) were recruited from 80 census block groups, using both active community outreach strategies (based on ethnographic information and interviews done with key informants) and passive methods (e.g. posting flyers in public places such as telephone poles, message boards inside of local bars, and message boards inside of some local stores). The census blocks chosen for inclusion in the study were selected based on neighborhood structural characteristics as reported in the 2000 U.S. Census Data and based on data from the Atlanta Police Department. Consistent with the study’s conceptual model and previous research findings [35,36], key neighborhood structural characteristics for inclusion were: (1) the percentage of household incomes that were reported to be more than 20% above or below the federal poverty level, (2) the percentage of adults who had not completed high school or its equivalent, (3) the percentage of female-headed households, (4) the percentage of people who were unemployed or not in the labor force, (5) the percentage of one-unit housing structures, (6) the percentage of owner-occupied households and (7) the percentage of vacant housing. Within the selected census block groups, the sampling frame was designed to ensure sufficient variability by gender, age (specifically, persons who were under the age of 35 and those aged 35 or older, to facilitate analytical comparisons based on younger versus older adults), and drug use (i.e., persons who had not used any illegal drugs during the previous 90 days versus those who had). In this context, a “drug user” was defined as someone who had used powder cocaine, crack cocaine, heroin, and/or methamphetamine at least once during the previous week and at least four times during the 90 days prior to interview. These criteria were imposed so that people designated as drug users for the purposes of this research were active, ongoing users of the illegal drugs in question, as opposed to one-time experimenters or casual, infrequent users of these substances. Conversely, in order to be considered a nonuser for this study, people had to report no use of these same four drugs during the five years prior to interview.

In order to be considered eligible for participation, respondents had to self-identify as African American, be at least 18 years of age, and have lived in that same neighborhood or census block group continuously for at least one year. People were considered ineligible for the study if they: (1) were in a drug treatment program or any other institutional setting at the time of recruitment, (2) were intoxicated at the time of consent or interview or (3) displayed signs of cognitive impairment at the time of consent or interview.

Computer-assisted structured interviews (CASI) were conducted with eligible persons in a private office that was located at a field site located in one of the catchment areas. The survey collected information about people’s demographic characteristics, psychological and psychosocial functioning, licit and illicit drug use history, sexual activity, criminal justice involvement and neighborhood perceptions. On average, interviews lasted approximately 90 min. The Emory University Institutional Review Board approved the study protocol.

### Measures

The principal measure of interest in this paper assesses respondents’ level of knowledge about HIV and its transmission. It is a summative scale measure comprised by responses to 12 items (Table 1) and was found to be reliable (Kuder-Richardson_20_=0.84). Compared to existing studies, we used a more nuanced approach than a straightforward “yes/no” or “true/false” response set for the items comprising the HIV knowledge scale. In recognition of the fact that many people make what they believe to be educated guesses about the safety or riskiness of engaging in various behaviors that they do not know for certain to be safe or risky, respondents in the People and Places study were allowed to give one of five responses to each HIV knowledge statement: “I know that it is true,” “I think that it is true,” “I think that it is false,” “I know that it is false,” and “I do not know whether the statement is true or false” (the latter being accepted only after the interviewer probed to make sure that people were not taking the easy way out of answering each specific knowledge item). In the construction of the scores of the HIV knowledge scale, the only answers that were counted as correct were those involving the “I know” option (because “I think” responses do not represent actual knowledge per se, but rather, guesses about the accuracy of a particular statement). Scores on the scale ranged from 0 to 12 (mean=5.22, SD=3.44), with higher scores indicating a greater level of knowledge about HIV and its transmission.

For the analysis focusing on the factors associated with greater/lesser levels of HIV knowledge, independent variables were selected from several domains hypothesized (based on previous research studies) to be related to HIV knowledge. Six demographic characteristics measures were examined: gender (male versus female), age (continuous), educational attainment (high school or less versus at least some college), sexual orientation (heterosexual versus gay, lesbian or bisexual), relationship status (“involved” with someone versus not “involved”), and religiosity (continuous). Childhood maltreatment experiences were assessed using six scale measures derived from Bernstein and Fink’s Childhood Trauma Questionnaire. All items asked respondents about their experiences prior to age 18 and included sexual abuse (Cronbach’s alpha=0.94), physical abuse (Cronbach’s alpha=0.77), emotional abuse (Cronbach’s alpha=0.81), physical neglect (Cronbach’s alpha=0.69), emotional neglect (Cronbach’s alpha=0.83), and overall amount of maltreatment (Cronbach’s alpha=0.91). Three items assessed respondents’ familiarity with persons infected with HIV: the number of HIV-infected persons known to the respondent (continuous), knowing anyone who is currently living with “full-blown” AIDS (yes/no) and knowing anyone who died from AIDS (yes/no). Five variables were used to examine substance use and abuse: the number of illegal drug types recently used (continuous), the frequency of recent alcohol use (continuous), the amount of recent alcohol use (continuous, computed by multiplying the number of days of use by the average number of drinks consumed per day), whether or not the person drank to the point of intoxication during the previous month (yes/no), and the number of alcohol problems experienced during the previous year (continuous scale measure, Kuder-Richardson_20_=0.82).

### Statistical analysis

Research Questions #1 and #2, pertaining to the extent of and gaps in HIV knowledge, are examined via the use of descriptive statistics. Research Question #3, pertaining to the factors associated with respondents’ amount of HIV knowledge, was undertaken in a two-step analytical process. In the first step, bivariate analyses were performed to determine which of the independent variables (listed above) were related to levels of HIV knowledge. Whenever the independent variable was dichotomous (e.g. gender, sexual orientation, recently became intoxicated), Student’s t tests were conducted. Whenever the independent variable was continuous in nature (e.g. amount of sexual abuse experienced, number of HIV-infected persons known, frequency of recent alcohol use), Pearson’s correlation coefficients (r) were computed.

In the second step, items identified as being related significantly (p<0.05) or marginally (0.10>p>0.05) to amount of HIV knowledge in the bivariate analyses were retained for entry into a multiple regression equation to determine which items were associated with the outcome measure when the effects of the other measures under consideration were taken into account. Both forward selection and backward elimination procedures were used to develop a fully reduced (or saturated) model containing only statistically significant predictors. The results obtained did not differ based on the selection/elimination approach used. Throughout all of these analyses, results are reported as being statistically significant whenever p<0.05.

## Results

### Sample characteristics

All participants in this study were African American and all lived in one of the specific Atlanta, Georgia census block groups selected for inclusion in this study. Slightly more than one-half (56.2%) of the respondents were male. Participants ranged in age from 18 to 92 (mean=37.3, SD=13.1). Overall, the respondents reported a fairly low level of educational attainment, with 39.1% indicating less than a complete high school education, 38.5% having completed high school or having earned a G.E.D., 20.2% having attended college but not completing it, and only 2.2% having graduated from college. Slightly more than one-half (55.6%) of the participants were married or involved with someone in a steady relationship at the time they took part in the study. Approximately 1 person in 14 (7.2%) self-identified as gay, lesbian or bisexual.

### Knowledge about HIV

Using the “traditional” approach to assessing participants’ levels of HIV knowledge (summing the number of items answered correctly on the true/false quiz, regardless of whether those correct answers were the result of knowing or guessing at the correct answer), overall levels of HIV knowledge appeared to be moderate, with people answering an average of nearly three-quarters (73.3%) of the knowledge items correctly (approximately 9 correct answers out of 12 questions) (SD=16.3). Even so, with HIV knowledge levels measured this way, very few respondents (3.9%) gave correct answers to all 12 of the HIV knowledge items. When HIV knowledge was assessed in this manner, a sizable majority of the participants gave correct answers to 6 of the 12 items: “A person can get HIV from a toilet seat” (85.2%), “Coughing and sneezing spread HIV” (81.1%), “It is possible to get HIV when a person gets a tattoo” (83.9%), “Pulling out the penis before a man comes keeps his partner from getting HIV during sex” (87.6%), “Showering or washing one’s genitals or private parts after sex keeps a person from getting HIV” (92.0%) and “You can usually tell if someone has HIV by looking at them” (85.1%).

An entirely different picture of HIV knowledge emerges, however, when we define knowledge only in terms of knowing the correct answer to each question rather than accepting accurate guesses as correct responses. When measured in this fashion, overall levels of actual HIV knowledge in this population were quite low. Table 1 presents the results for the individual items. The average score on the HIV knowledge quiz items was 43.5% (approximately 5 correct answers out of 12 questions) (SD=28.6) and only 1.2% of the study participants gave correct answers to all 12 questions. In fact, for only 3 of the 12 items examined did even a simple majority of the respondents provide correct answers. These items were: “Pulling out the penis before a man comes keeps his partner from getting HIV during sex” (53.6%); “Showering or washing one’s genitals or private parts after sex keeps a person from getting HIV” (62.9%); and “You can usually tell if someone has HIV by looking at them” (61.6%). Conversely, substantial majorities of the respondents gave incorrect answers to 3 of the 12 items as well: “There is a vaccine that can stop adults from getting HIV” (61.8%); “There is a cure for AIDS” (77.0%); and “HIV is killed by cleaning a syringe multiple times with bleach and water before using it again” (91.9%).

Throughout the remainder of this paper, whenever we refer to respondents’ levels of HIV knowledge, we are referring to the assessment method that is based on their actual knowledge rather than the combination of correct guesses and known answers to the items in question.

### Factors associated with greater HIV knowledge

Several of the demographic characteristics measures differentiated respondents based on their levels of HIV knowledge. As age increased, people’s levels of HIV information decreased (p<0.001). People who had no more than a high school education scored more poorly on the HIV knowledge items than their better-educated counterparts did (41.4% versus 50.7%; p<0.001). Respondents who self-identified as heterosexual were lower in their levels of HIV knowledge when compared to their gay, lesbian, and bisexual counterparts (42.7% versus 53.0%; p<0.001). HIV knowledge did not differ based on gender, religiosity, or relationship status.

All but one of the childhood maltreatment experiences measures differentiated study participants based on their levels of HIV knowledge. Counterintuitively, the more sexual abuse (p<0.001), physical abuse (p<0.001), emotional abuse (p<0.001), emotional neglect (p<0.05) or overall childhood maltreatment (p<0.001) that people experienced during their formative years, the higher they tended to score on the HIV knowledge items. Differences in HIV knowledge levels were not found based on physical neglect.

Two of the three measures assessing familiarity with people who have HIV also were related to HIV knowledge levels. The more HIV-infected people the respondent knew, the better he/she tended to score on the HIV knowledge quiz (p<0.001). Similarly, participants who knew someone currently living with full-blown AIDS did better on the HIV knowledge quiz than participants who did not know any such person(s) (46.9% versus 41.6%; p<0.001). In contrast, knowing versus not knowing anyone who previously had died from AIDS was found to be unrelated to HIV knowledge scores.

Two of the substance use/abuse measures examined were found to be related to respondents’ HIV knowledge levels. The more frequently that people used alcohol, the less they tended to know about HIV (p=0.016). Additionally, the larger the number of different types of drugs the person had used during the 90 days prior to interview, the poorer he/she tended to score on the HIV knowledge quiz (p=0.002). Knowledge about HIV was not found to be related to: the amount of alcohol that respondents consumed in the month prior to interview, whether or not the person had drunk alcohol to the point of intoxication during the month prior to interview or the number of alcohol-related problems the person had experienced during the previous year.

Next, the items that were statistically significant or marginally significant in the bivariate analyses (above) were entered into a multivariate equation, to determine which ones were associated with HIV knowledge levels when the effects of the other measures were taken into account. Seven items were found to contribute uniquely and significantly to greater levels of HIV knowledge (Table 2). These were: (1) age (p<0.001), (2) having no more education than a high school diploma (p<0.001), (3) self-identifying as gay, lesbian, or bisexual (p<0.05), (4) having been sexually abused during childhood and/or adolescence (p<0.05), (5) less frequent recent use of alcoholic beverages (p<0.05), (6) knowing a larger number of people who were infected with HIV (p<0.001) and (7) knowing anyone who is currently living with full-blown AIDS (p<0.01). Together, these seven items explained 6.3% of the total variance.

### Interaction effects

**Figure 1:**
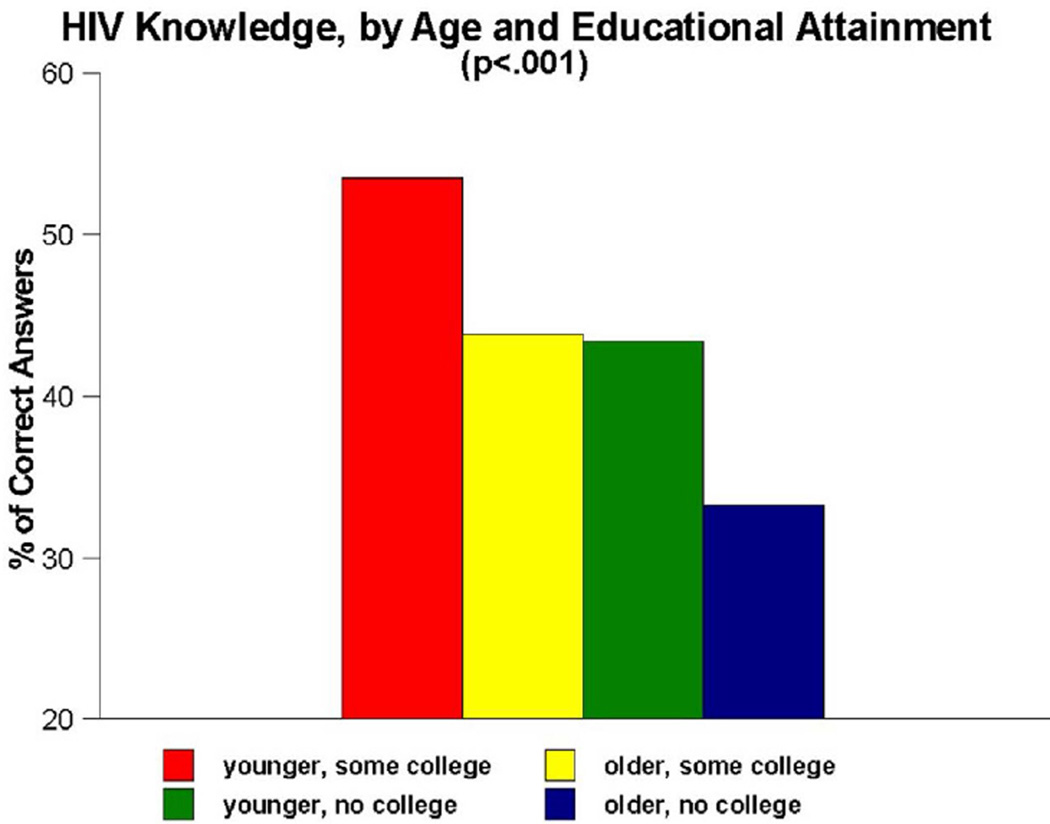
HIV knowledge, by age and educational attainment.

As Figure 1 demonstrates, important interaction effects were observed for age and educational attainment (p<0.001). Post hoc analyses of the age measure suggested a divide in HIV knowledge between persons aged 18 to 49 and those aged 50 and older. Thus, a four-category interaction measure was created to examine differences amongst (A) younger adults who had at least some college education, (B) older adults who had at least some college education, (C) younger adults whose educational attainment was no greater than a high school diploma and (D) older adults whose educational attainment was no greater than a high school diploma. Paired-comparisons tests showed that group A scored significantly better than all other groups on the HIV knowledge items (53.5% versus 43.9%, 43.4% and 33.3%, respectively), while group D scored significantly worse than all other groups.

**Figure 2:**
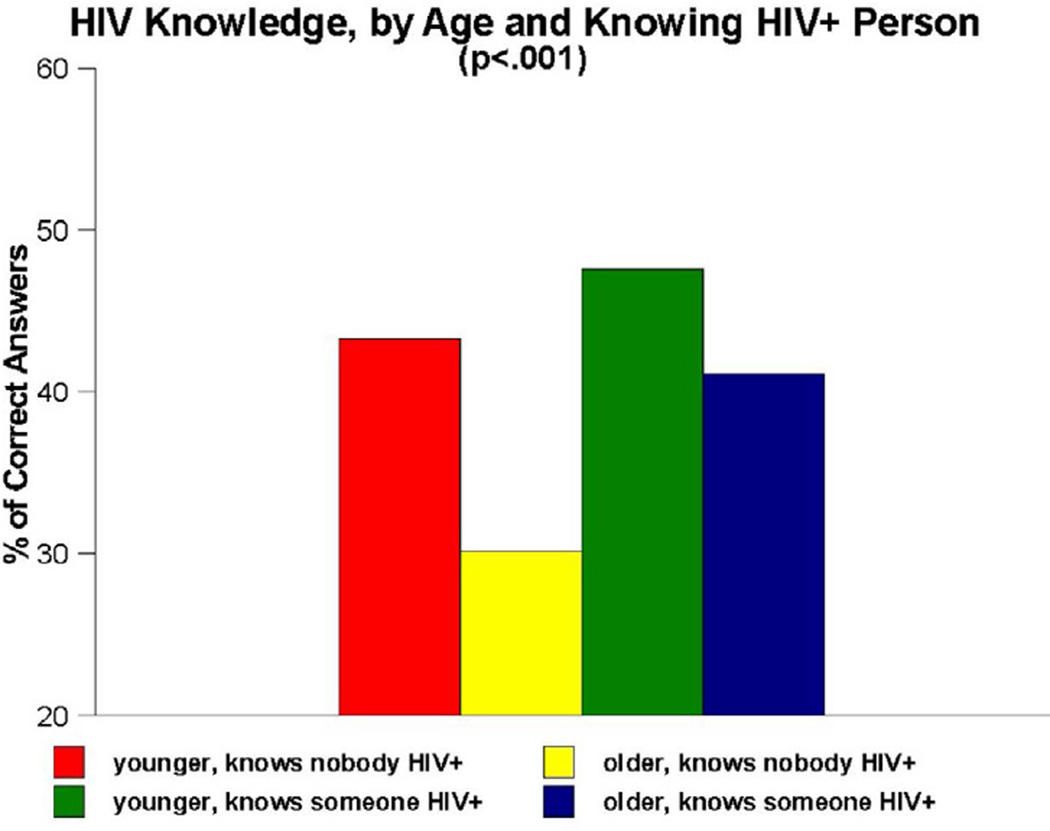
HIV knowledge, by age and knowing anyone who is HIV-infected.

Important interaction effects were also observed for age and knowing versus not knowing anyone who is HIV-infected (p<0.001) (Figure 2). Once again, age was divided into persons aged 18 to 49 and those aged 50 and older. Another four-category interaction measure was created, this time to examine differences amongst (A) younger adults who knew nobody who was HIV-positive, (B) older adults who knew nobody who was HIV-positive, (C) younger adults who knew at least one HIV-infected person, and (D) older adults who knew at least one HIV-infected person. Paired-comparisons tests revealed that group C scored significantly higher on the HIV knowledge items than all other groups (47.6% versus 43.3%, 30.2% and 41.2%, respectively), whereas group B scored significantly lower than all other groups.

**Figure 3:**
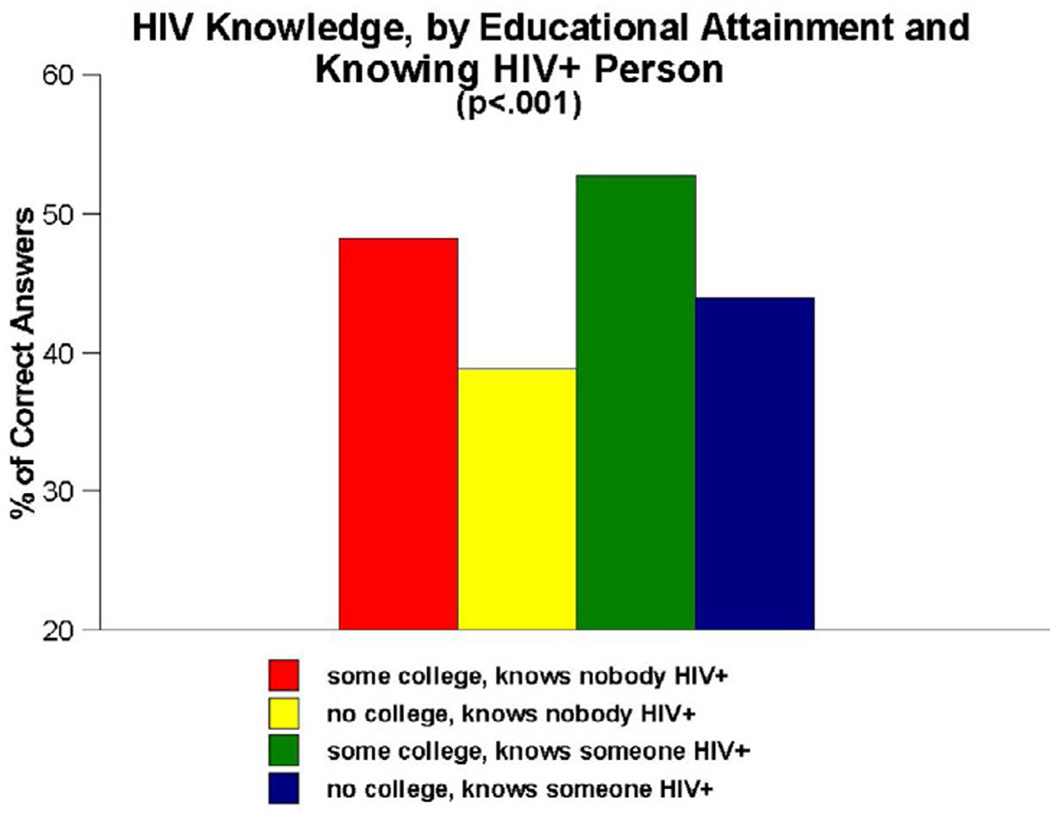
HIV knowledge, by educational attainment and knowing anyone who is HIV-infected.

As Figure 3 depicts, noteworthy interaction effects were also obtained for the combination of educational attainment and knowing versus not knowing anyone who is HIV-infected (p<0.001). Here, the four-way interaction measure compared the following groups: (A) people who had at least attended college and who knew nobody who was HIV-positive, (B) people who had no more than a high school education and who knew nobody who was HIV-positive, (C) people who had at least attended college and who knew at least one HIV-infected person and (D) people with no more than a high school education and who knew at least one HIV-infected person. The paired-comparisons tests revealed that group B scored more poorly on the HIV knowledge items than persons in all other groups (38.9% versus 48.2%, 52.7% and 43.9%, respectively).

**Figure 4:**
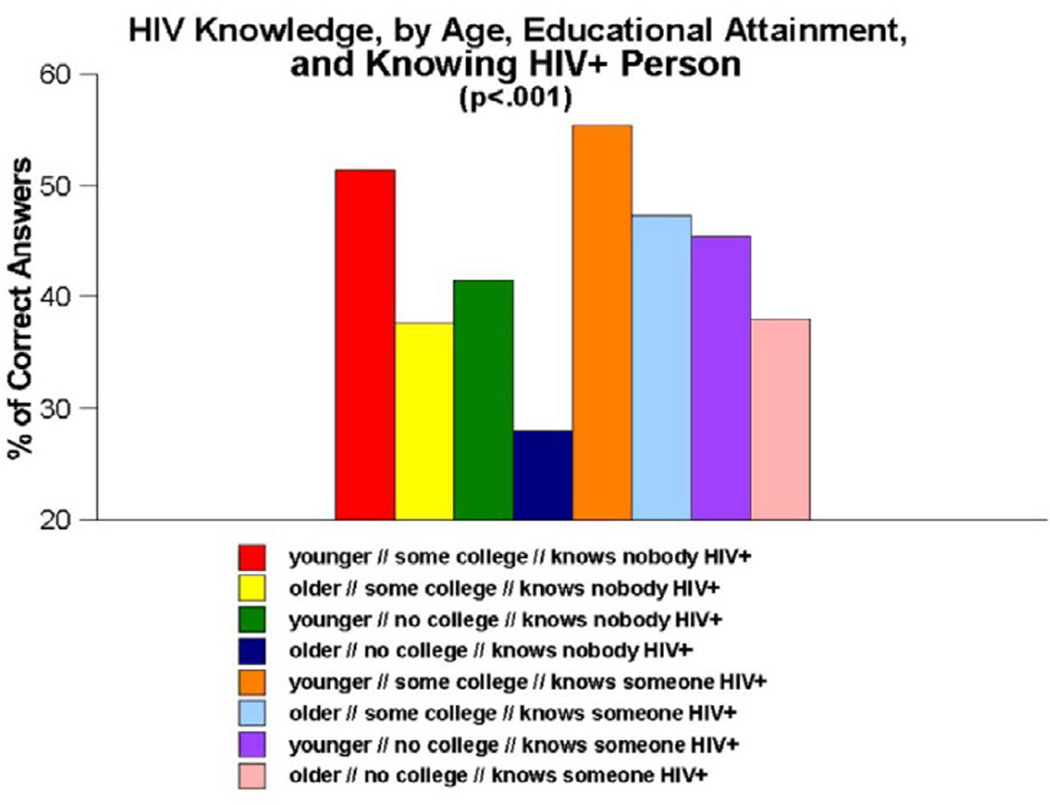
HIV knowledge, by age, educational attainment and knowing anyone who is HIV-infected.

When all three of these measures are combined into a three-way interaction measure to examine the conjoint effects of age, educational attainment, and knowing versus not knowing anyone who is infected with HIV (Figure 4), once again, a significant main effect was observed for levels of HIV knowledge (p<0.001). Paired-comparisons tests showed that one group stood out from all others–namely, older adults with no more than a high school education who knew nobody who was HIV-infected. These persons scored more poorly than all other groups, with an average correct score of 27.9% on the HIV knowledge quiz. In contrast, knowing significantly more about HIV than all groups but one were the younger adults with at least some college education who knew at least one person who was HIV-positive. These individuals performed twice as well as the previously discussed group, with an average score of 55.4% correct answers.

## Discussion

### Limitations of this research

The authors would like to acknowledge a few potential limitations of this research. First, it was conducted with a sample of African Americans residing in a major metropolitan area. Persons living in other environments that are less densely populated may not share the same life and community experiences as those living in urban areas such as the one where the present study was conducted. Additionally, this research was conducted in the American South. African Americans living in other parts of the country may have different socio-ecological experiences compared to the persons who participated in the People and Places study. The extent to which these geographic factors affected the present study’s findings is not known and cannot be assessed with the available data.

Second, all data for the People and Places study were based on uncorroborated self-reported behaviors. The extent to which reliance upon self-reported data may have affected this study’s findings cannot be assessed with the available data. In all likelihood, the self-reported data can be trusted, as numerous authors have noted that persons in their research studies (which, like the present study, have included fairly large numbers of substance abusers and/or persons at risk for contracting or transmitting HIV) have provided accurate information about their behaviors [37–39].

A third potential limitation comes in the form of the specific HIV/AIDS knowledge questions asked. Although the items selected for use in the People and Places study were all fairly straightforward in nature, it is possible that the addition of more knowledge-related questions and/or the substitution of the questions that were used with others that were not used could have led to different assessments of respondents’ overall levels of HIV-related knowledge. There is no “gold standard” of HIV/AIDS knowledge questions currently in use and, likewise, there is no agreed-upon HIV/AIDS knowledge inventory that has been shown to be superior to others for assessing various populations’ actual knowledge about HIV and how it is/not transmitted. Consequently, there is no way of knowing how, if at all, using different knowledge-related questions than those employed in the People and Places study might have affected the findings obtained in this research.

## Conclusion

Despite these potential limitations, the authors still believe that the present research has much to offer to professionals working in the HIV prevention and intervention fields. To begin with, the way in which we measure the amount of HIV knowledge that people have makes a fairly consequential difference in terms of how much knowledge we can ascribe to them. In the present study, when a traditional approach to assessing HIV knowledge was used–one that relied upon a simple true/false schema for replying to the individual questions–respondents in this particular study population scored in the moderate to moderately high range, averaging 73.3% correct answers (or approximately 9 out of the 12 knowledge items used). When a more-nuanced approach was used, however–one that distinguished respondents’ actual knowledge from their ability to guess correctly at the answers to knowledge items about which they were uncertain–a very different picture emerged: Knowledge scores dropped noticeably, to 43.5% correct answers (or approximately 5 of the 12 knowledge items used). When HIV knowledge was assessed such that it was based only on knowing, rather than knowing or guessing, the respondents possessed, at best, fairly low levels of knowledge regarding the transmission of HIV. Future researchers who are interested in assessing levels of HIV knowledge, and those who wish to determine whether a particular HIV educational program or intervention initiative has led to increases on participants’ knowledge about HIV transmission, could benefit from the present research by considering the adoption of improved, nuanced methods of evaluating HIV knowledge such as that employed in the present study.

Regardless of which method of assessing HIV/AIDS knowledge is employed–the “traditional” approach using a simple true/false schema to determine overall level of knowledge or our recommended “modified” or “nuanced” approach that differentiates amongst answers based on actual knowledge versus a “best guesses” approach to answering–the present study found that HIV/AIDS knowledge rates were far less than optimal in our research population. Moreover, on several of the specific knowledge items used in this study–item generally considered representing what should be common knowledge in terms of contemporary public health education pertaining to HIV/AIDS–sizable proportions of the study participants did not know the correct answers. From a public health perspective and from an HIV prevention standpoint, this raises profound concerns regarding the gaps in HIV knowledge in this population. Undoubtedly, at least some of the much-higher-than-average rates of HIV and other STI transmission that have been identified in this study population can be attributed to a lack of knowledge about the riskiness of engaging in certain behaviors. Although previous research has demonstrated neither a strong nor a consistent link between HIV/AIDS knowledge and involvement in risky behaviors [30–34], having an adequate comprehension of just what practices do and do not constitute behavioral risk for contracting/transmitting HIV and other STIs is a necessary component of any effective HIV risk reduction program. That is, whilst HIV knowledge has not been shown to be necessary and sufficient in the determination of people’s involvement in risky practices, it is nonetheless a necessary element thereof. Quite simply, we cannot reasonably expect people to endeavor to change behaviors that they do not know are risky. Thus, one of the most important findings obtained in the present study is the determination of just how low our study participants’ overall levels of HIV/AIDS knowledge were, because this highlights the importance of developing improved strategies for informing urban-dwelling African Americans in the South (and probably those residing in other parts of the United States as well, given their higher-than-average rates of HIV infection) about how HIV is/not transmitted and what, exactly, they can do to reduce their risk for contracting or transmitting HIV. Mays and colleagues developed a thoughtful chapter addressing the issues surrounding and the challenges pertaining to HIV prevention among African American women in the South. Additionally, Zuniga et al. provided a summary of outreach and intervention programs, as well as outcomes obtained and challenges faced by those programs, for 222 community-based HIV/AIDS service organizations in the Deep South [40]. Both of the aforementioned works are well worth consulting for readers who are interested in learning more about these subjects.

The implication of our low-knowledge finding is particularly troubling, we believe, when one considers the sheer number of community-based efforts that have been undertaken in recent years specifically to target HIV risk practices and/or to improve HIV knowledge among members of the African American community (and various subpopulations thereof) in the American South. Zule et al. reported positive findings for their North Carolina-based HIV intervention project targeting African Americans (as well as people of other racial/ethnic groups) visiting local-area health departments and women’s centers [41]. Wingood et al. also obtained positive outcomes (particularly consistent condom usage) in their Georgia-based HIV intervention work targeting young adult female African Americans who were recruited via an African American church [42]. In a separate study focusing on African American women aged 18–29 who were recruited from Atlanta-area insurance health care agencies, Wingood et al. intervention yielded results indicating lower rates of new STIs, higher rates of condom use, and lower rates of having concurrent sex partners [43]. Aronson et al. conducted their HIV intervention project with African American male college students located at two universities in North Carolina and found that men in their intervention condition reported fewer instances of improper condom use and reduced rates of unprotected sex compared to baseline [44]. MacMaster et al. implemented their HIV intervention project in Tennessee with African American substance abusers, and at follow-up discovered reductions in participants’ number of recent sex partners, number of times having condom less sex, and number of times engaging in sex trading activities [45]. Diallo et al. conducted their HIV/STI intervention work with Atlanta, Georgia-area African American women who were recruited in church settings, colleges and community centers, and found that intervention participants were more likely than those in the control condition to report recent condom use during sex and to have had a recent HIV test [46]. Literally dozens of other community-based projects have been implemented with similar kinds of success throughout the South, targeting other subpopulations of African Americans. Yet despite all of the favorable outcomes obtained by these studies and community-based projects, HIV knowledge levels still remain low among Southern African Americans and HIV infection rates still remain high. There seems to exist a disparity between the main findings of community-based initiatives designed to reduce specific aspects of HIV risk taking in various African Americans of the South and actual levels of HIV knowledge among participants (and nonparticipants) in those studies.

Another important finding generated by the present research was that, although most participants in the People and Places study were insufficiently informed about HIV, certain subgroups of participants were even less informed than others. Our interaction analyses–which focused on the impact of age, educational attainment and knowing versus not knowing anyone who was infected with HIV–revealed that all three of these measures interacted with one another, both when they were examined in pairs of measures and when all three measures were examined conjointly, in such a manner as to heighten the impact on HIV knowledge. Being older (i.e., aged 50 or older), having no more than a high school education, and not knowing anyone who had been infected with HIV were all associated with having lower levels of HIV knowledge in this population. These effects were particularly noteworthy, as Figures 1–4 all show, when the effects of these measures were examined in combination with one another. The combined impact of age, educational attainment, and familiarity with someone who is HIV-infected on our respondents’ levels of HIV knowledge suggest that syndemic-type effects are in operation here. Walkup et al. noted that health problems may be construed as syndemic when two or more conditions/afflictions are linked in such a manner that they interact synergistically, with each contributing to an excess burden of disease in a particular population [47]. In the present study, the syndemic effects appear to be the result of how two demographic traits (age, educational attainment) and one life experience measure (familiarity with HIV-infected persons) interplay and heighten the impact of one another in terms of their influence on HIV knowledge. Numerous researchers have written about the importance of utilizing a syndemics theory approach to studying HIV risk factors [48–52]. The present study is consistent with those reports and importantly, expands upon them by demonstrating another application of the main principles of syndemics theory to a somewhat different population (namely, urban Southern-dwelling African Americans) and a slightly different outcome measure (namely, HIV knowledge) than have been used in the scientific literature published to date.

Finally, we would like to discuss the implications of some of the more important findings of our multivariate analysis, which revealed several characteristics that were associated with levels of HIV knowledge. The first of these was age, with younger respondents being more knowledgeable about HIV and its transmission than older respondents were. This finding was particularly pronounced when the knowledge comparison was made between persons aged 18 to 49 versus those aged 50 or older. Other researchers have also demonstrated lower levels of HIV knowledge among older persons [53–55] and the present research is consistent with those studies. Several factors appear to contribute to this finding. First, many older adults perceive themselves to be invulnerable to the effects of HIV, thereby “tuning out” HIV educational messages to which they are exposed. Second, most HIV/AIDS educational and prevention programs have tended not to target their messages at older audiences, or to provide older adults with age-specific educational campaigns where HIV/AIDS are concerned [56,57]. As a result, many older adults may feel uncomfortable attending HIV education, prevention and/or intervention programs that are populated principally by younger persons, for fear of “standing out in a crowd” or being unwelcomed by people who are young enough to be their children or grandchildren. Third, research has shown that professionals in the health care industry (e.g. physicians, nurses, social workers) who have regular contact with older adults typically do not speak with these persons about such matters as sexual practices and sexual safety [57,58]. This has been attributed to the health care professionals experiencing some level of discomfort talking about a sensitive, personal, private matter such as sex with people who are their elders, as well as denial on the part of many health care professionals with respect to the sexual activities of their older patients/clients. Regardless of its cause(s), the implication of our age-related finding is clear: HIV educational initiatives and intervention programs need to target older adults and to find effective ways of reaching and teaching older African Americans about the various ways that HIV can/not be transmitted.

In our multivariate analysis, we also found that HIV knowledge was greater among people who had attended college when compared to those who had not. This finding is consistent with other published reports [59,60] and it indicates a two-fold need: First, it suggests that persons with lower levels of education ought to be targeted by HIV educational and intervention programs, because they more than others are likely to be lacking in knowledge about how HIV is transmitted. This is particularly important when considering racial disparities in educational attainment [61]. Second, our education-related finding also suggests that targeted HIV initiatives for this population may have to be especially careful with regard to making their messages accessible to people with lower levels of education, comprehension, and/or literacy. Other researchers, as well, have addressed the need to give special consideration to issues surrounding low levels of health literacy when providing HIV education [62].

Our multivariate analysis also revealed an inverse relationship between frequency of alcohol consumption and HIV knowledge level. Numerous published reports have demonstrated a link between alcohol consumption and involvement in various types of HIV risk practices among various subpopulations of African Americans [63–65]. Our finding is consistent with these studies’ findings, yet still expands upon them by documenting a link between alcohol use and knowledge about HIV. It suggests that HIV intervention efforts ought to continue to target substance abusers, particularly those African Americans who drink the most frequently, as this group represents an “at risk” population. Over the years, several authors have written about the value of incorporating HIV education and prevention services into existing substance abuse treatment programs [66,67]. The present study’s findings suggest that this might be a beneficial approach to helping to improve HIV transmission knowledge in a population that is greatly in need of such information.

Finally, the present study’s multivariate analysis also demonstrated that familiarity with persons who were living with HIV or with so-called full-blown AIDS was associated with greater levels of knowledge about HIV. We believe that this finding indicates that having acquaintances, friends and/or relatives who are experiencing the effects of being infected with HIV leads some people to engage in conversations with these HIV-infected others in their life about how they initially became infected with the virus, what they might have been able to do differently, and how others who are not infected with HIV can remain HIV-negative. One plausible application of this particular research finding would be for community-based HIV education, prevention, and intervention initiatives to utilize the personal experiences of HIV-infected persons as part of the delivery of their risk reduction messages. This type of “each one, reach one, teach one” approach to HIV education and prevention–sometimes referred to as a peer-led approach–not only personalizes the information and messages that are being provided by the program, but also gives credibility to the intervention content being offered. Several such programs exist around the United States, such as New York City’s Each One Reach One, San Francisco’s Keeping It Safe, New Castle, Delaware’s Each One Teach One, and Washington, DC’s Saving Our Sisters from HIV/AIDS, among numerous others. This approach has also been shown to be an effective way of achieving desired outcomes [68,69]. In the context of the present study’s findings, we believe that enhancing HIV knowledge among urban-dwelling African Americans in the South might be something effectively accomplished through the implementation of well-trained peer networks, in which already-infected persons conduct community outreach and educational/intervention sessions.

## Figures and Tables

**Table 1:** Knowledge about HIV.

HIV Knowledge Item	Know It is True	Think It is True	Think It is False	Know It is False	Don’t Know
There is a cure for AIDS.	11.7	42.6	22.2	20.1	0.5
A person can get HIV from a toilet seat.	2.2	12.2	36.0	49.2	0.4
Coughing and sneezing spread HIV.	2.4	16.1	32.7	48.4	0.4
A person can get HIV by sharing a glass of water with someone who has HIV.	6.2	21.2	30.5	41.5	0.6
HIV is killed by cleaning a syringe multiple times with bleach and water before using it again.	8.1	15.8	37.3	37.4	1.5
It is possible to get HIV when a person gets a tattoo.	44.5	39.4	10.6	4.7	0.8
Pulling out the penis before a man comes keeps his partner from getting HIV during sex.	2.7	9.1	34.1	53.6	0.6
Showering or washing one’s genitals or private parts after sex keeps a person from getting HIV.	1.8	5.8	29.2	62.9	0.3
People who have been infected with HIV quickly show serious signs of being infected.	7.3	15.7	29.1	47.4	0.5
There is a vaccine that can stop adults from getting HIV.	4.8	21.0	35.0	38.2	1.0
You can usually tell if someone has HIV by looking at them.	4.6	10.0	23.6	61.6	0.3
Taking a test for HIV one week after having sex will tell a person if he or she has HIV.	6.8	18.2	30.8	43.4	0.8

**Table 2:** Factors associated with greater level of HIV knowledge.

Independent Variable	b	β	p=|x|
Age	−0.003	0.15	<0.001
Educational Attainment=High School or Less	−0.095	0.14	<0.001
Sexual Orientation=Gay/Bisexual	0.065	0.06	0.012
Amount of Sexual Abuse Experienced	0.016	0.06	0.014
Frequency of Recent Alcohol Consumption	−0.001	0.05	0.031
Number of HIV+ People Respondent Knows	0.003	0.08	<0.001
Respondent Knows Someone Living with AIDS	0.042	0.05	0.003
